# Supplementary material for: Mitochondrial fatty acid synthesis coordinates oxidative metabolism in mammalian mitochondria
Source: eLife. 2020 Aug 17;9:e58041. doi: 10.7554/eLife.58041 (PMC7470841; doi:10.7554/eLife.58041)
Supplement: Supplementary file 1. — All steady-state metabolites measured via LCMS. Values shown are average fold change from mean GFP abundance. [file elife-58041-supp1.docx]

**Supplementary File 1:**

| **Metabolite** | **GFP-1** | **Mcat-1** | **Oxsm-1** | **Mecr-1** |
| --- | --- | --- | --- | --- |
| **OXOGLUTARATE** | 1.000 | 14.342 | 22.874 | 16.937 |
| **GLUTATHIONE REDUCED** | 1.000 | 2.590 | 9.888 | 5.128 |
| **OXIDIZED GLUTATHIONE** | 1.000 | 3.229 | 7.622 | 4.521 |
| **2-HYDROXYGLUTARIC** | 1.000 | 4.121 | 4.670 | 2.290 |
| **INOSINE** | 1.000 | 0.797 | 3.535 | 0.521 |
| **HYPOXANTHINE** | 1.000 | 1.303 | 3.394 | 0.771 |
| **DEOXYGUANOSINE-MONOPHOSPHATE** | 1.000 | 1.437 | 3.118 | 1.101 |
| **BETAINE ALDEHYDE** | 1.000 | 2.179 | 3.085 | 3.982 |
| **6-HYDROXYDOPAMINE** | 1.000 | 2.085 | 3.051 | 3.791 |
| **N-METHYLGLUTAMATE** | 1.000 | 2.995 | 2.845 | 3.340 |
| **OXOADIPATE** | 1.000 | 3.345 | 2.832 | 2.380 |
| **NICOTINAMIDE MONONUCLEOTIDE** | 1.000 | 0.981 | 2.782 | 1.168 |
| **AMINOADIPATE** | 1.000 | 2.086 | 2.649 | 2.014 |
| **PYRUVATE** | 1.000 | 2.641 | 2.318 | 3.455 |
| **GUANOSINE MONOPHOSPHATE** | 1.000 | 0.954 | 2.102 | 0.860 |
| **CYSTEINE** | 1.000 | 1.032 | 1.949 | 1.620 |
| **2-KETOHEXANOIC** | 1.000 | 2.995 | 1.822 | 1.308 |
| **Fructose 1,6-bisphosphate** | 1.000 | 0.911 | 1.818 | 2.217 |
| **URATE-2** | 1.000 | 4.273 | 1.817 | 1.944 |
| **URATE-1** | 1.000 | 4.273 | 1.816 | 1.944 |
| **ADENOSINE-MONOPHOSPHATE** | 1.000 | 0.979 | 1.804 | 0.632 |
| **DIETHANOLAMINE** | 1.000 | 1.141 | 1.725 | 1.052 |
| **3-METHYL-2-OXOVALERATE** | 1.000 | 2.918 | 1.686 | 1.125 |
| **DL-2-AMINOCTANOATE** | 1.000 | 0.876 | 1.662 | 1.456 |
| **KETOLEUCINE** | 1.000 | 2.873 | 1.655 | 1.110 |
| **KYNURENINE** | 1.000 | 1.979 | 1.650 | 1.300 |
| **4-GUANIDINOBUTANOATE-2** | 1.000 | 1.682 | 1.567 | 1.231 |
| **TRIMETHYLAMINE** | 1.000 | 1.844 | 1.538 | 1.408 |
| **URIDINE MONOPHOSPHATE** | 1.000 | 1.313 | 1.536 | 1.289 |
| **AICAR** | 1.000 | 0.721 | 1.532 | 1.190 |
| **N,N-DIMETHYLARGININE** | 1.000 | 1.967 | 1.532 | 1.621 |
| **Octanoate** | 1.000 | 1.231 | 1.496 | 1.498 |
| **LPC(18:1)** | 1.000 | 2.071 | 1.458 | 1.226 |
| **CYTIDINE MONOPHOSPHATE** | 1.000 | 1.318 | 1.449 | 0.670 |
| **4-HYDROXY-3-METHOXYPHENYLGLYCOL** | 1.000 | 1.450 | 1.446 | 1.798 |
| **SPERMIDINE** | 1.000 | 1.247 | 1.446 | 1.169 |
| **3-Hydroxysebacic acid** | 1.000 | 1.244 | 1.425 | 1.369 |
| **OXOPROLINE** | 1.000 | 2.313 | 1.402 | 1.466 |
| **hexanoylcarnitine** | 1.000 | 1.220 | 1.395 | 1.842 |
| **MANDELATE** | 1.000 | 0.865 | 1.390 | 1.545 |
| **2-HYDROXYPHENYLACETATE** | 1.000 | 1.067 | 1.377 | 1.545 |
| **CYTIDINE** | 1.000 | 3.482 | 1.341 | 1.308 |
| **PALMITATE** | 1.000 | 0.968 | 1.336 | 1.284 |
| **LPC(16:0)** | 1.000 | 2.650 | 1.317 | 1.425 |
| **6-Phosphonoglucono-D-lactone** | 1.000 | 0.645 | 1.302 | 2.068 |
| **HYDROXYISOCAPROIC** | 1.000 | 1.292 | 1.298 | 1.497 |
| **MANNOSE 6-PHOSPHATE** | 1.000 | 0.527 | 1.298 | 1.507 |
| **ACETOIN** | 1.000 | 1.245 | 1.288 | 1.221 |
| **GLUCOSE 6-PHOSPHATE** | 1.000 | 0.543 | 1.274 | 1.515 |
| **GLUCOSE 1-PHOSPHATE** | 1.000 | 0.541 | 1.273 | 1.513 |
| **FRUCTOSE 6-PHOSPHATE** | 1.000 | 0.541 | 1.273 | 1.513 |
| **D-RIBOSE 5-PHOSPHATE** | 1.000 | 0.786 | 1.268 | 1.366 |
| **GLUTARATE** | 1.000 | 1.238 | 1.263 | 1.323 |
| **DEOXYCYTIDINE-DIPHOSPHATE** | 1.000 | 0.531 | 1.259 | 0.297 |
| **SEDOHEPTULOSE-7-PHOSPHATE** | 1.000 | 0.439 | 1.244 | 1.333 |
| **BETA-GLYCEROPHOSPHATE** | 1.000 | 1.207 | 1.231 | 1.082 |
| **RIBULOSE-5-PHOSPHATE** | 1.000 | 0.754 | 1.229 | 1.325 |
| **URIDINE 5'-DIPHOSPHATE** | 1.000 | 1.344 | 1.229 | 1.316 |
| **Ribose-5-phosphate** | 1.000 | 0.756 | 1.227 | 1.326 |
| **Xylulose-5-phosphate** | 1.000 | 0.751 | 1.225 | 1.323 |
| **7-HYDROXYOCTANOATE** | 1.000 | 1.124 | 1.224 | 1.242 |
| **SPERMINE** | 1.000 | 0.521 | 1.222 | 1.276 |
| **undecanedioic acid** | 1.000 | 1.140 | 1.219 | 1.273 |
| **GLYCEROL 3-PHOSPHATE** | 1.000 | 1.194 | 1.218 | 1.052 |
| **LEUCINE** | 1.000 | 0.958 | 1.217 | 0.915 |
| **METHYL 4-AMINOBUTYRATE** | 1.000 | 1.828 | 1.215 | 1.137 |
| **O-SUCCINYL-HOMOSERINE-2** | 1.000 | 0.993 | 1.200 | 1.410 |
| **SUBERATE** | 1.000 | 1.093 | 1.194 | 1.226 |
| **Dimethyl adipate** | 1.000 | 1.092 | 1.193 | 1.213 |
| **PROPIONYLCHOLINE** | 1.000 | 1.185 | 1.190 | 1.270 |
| **2-ETHYLOCTANEDIOATE** | 1.000 | 1.139 | 1.190 | 1.254 |
| **SEBACATE** | 1.000 | 1.139 | 1.190 | 1.254 |
| **DEOXYCYTIDINE MONOPHOSPHATE-1** | 1.000 | 1.017 | 1.188 | 1.255 |
| **DEOXYINOSINE** | 1.000 | 0.988 | 1.186 | 1.095 |
| **6-CARBOXYHEXANOATE** | 1.000 | 1.116 | 1.169 | 1.241 |
| **L-CARNITINE** | 1.000 | 1.049 | 1.163 | 1.526 |
| **Azelaic acid** | 1.000 | 1.173 | 1.161 | 1.209 |
| **Butyrylcarnitine** | 1.000 | 1.180 | 1.161 | 1.536 |
| **CREATINE** | 1.000 | 1.012 | 1.158 | 1.099 |
| **GLYCOLATE** | 1.000 | 0.625 | 1.152 | 2.708 |
| **4-HYDROXYBENZOATE** | 1.000 | 1.049 | 1.143 | 0.998 |
| **LACTATE** | 1.000 | 1.412 | 1.142 | 1.397 |
| **AZELATE** | 1.000 | 1.145 | 1.142 | 1.179 |
| **THYMIDINE** | 1.000 | 1.258 | 1.141 | 1.086 |
| **SALICYLATE** | 1.000 | 1.020 | 1.136 | 1.235 |
| **MONOMETHYLGLUTARATE** | 1.000 | 1.171 | 1.135 | 1.184 |
| **NICOTINAMIDE-1** | 1.000 | 1.777 | 1.124 | 1.248 |
| **GLYCERALDEHYDE** | 1.000 | 1.405 | 1.123 | 1.376 |
| **P-HYDROXYPHENYLACETATE** | 1.000 | 0.700 | 1.123 | 1.140 |
| **2-METHYLGLUTARATE** | 1.000 | 1.133 | 1.118 | 1.176 |
| **METHYGLUTARATE** | 1.000 | 1.166 | 1.118 | 1.181 |
| **N-ACETYLNEURAMINATE** | 1.000 | 1.755 | 1.114 | 0.815 |
| **ADIPATE** | 1.000 | 1.052 | 1.113 | 1.131 |
| **LYSINE** | 1.000 | 1.734 | 1.110 | 1.025 |
| **4-HYDROXYPHENYLPYRUVATE** | 1.000 | 1.348 | 1.105 | 0.792 |
| **3-HYDROXYMETHYLGLUTARATE** | 1.000 | 1.349 | 1.099 | 0.966 |
| **DEOXYGUANOSINE** | 1.000 | 1.168 | 1.097 | 1.162 |
| **BUTANOATE** | 1.000 | 1.524 | 1.089 | 1.083 |
| **4-HYDROXYBENZALDEHYDE** | 1.000 | 1.347 | 1.080 | 0.762 |
| **PANTOTHENATE** | 1.000 | 2.106 | 1.078 | 0.682 |
| **THYMIDINE-MONOPHOSPHATE** | 1.000 | 0.800 | 1.072 | 0.607 |
| **NAD+** | 1.000 | 1.270 | 1.068 | 1.022 |
| **octanoylcarnitine** | 1.000 | 1.324 | 1.058 | 1.234 |
| **DEOXYCYTIDINE-1** | 1.000 | 1.612 | 1.056 | 0.882 |
| **PYRAZOLE** | 1.000 | 1.213 | 1.054 | 0.824 |
| **SACCHARATE** | 1.000 | 0.844 | 1.049 | 0.988 |
| **7-HYDROXYOCTANOATE DIMER** | 1.000 | 1.068 | 1.047 | 1.003 |
| **GLYCEROPHOSPHOCHOLINE** | 1.000 | 2.345 | 1.039 | 0.870 |
| **URIDINE** | 1.000 | 1.203 | 1.036 | 1.257 |
| **PYROGLUTAMATE** | 1.000 | 2.255 | 1.034 | 2.031 |
| **HYPOTAURINE** | 1.000 | 0.559 | 1.033 | 1.901 |
| **6-PHOSPHOGLUCONATE** | 1.000 | 0.661 | 1.033 | 1.166 |
| **TAURINE** | 1.000 | 0.689 | 1.031 | 1.444 |
| **N-METHYLASPARTATE** | 1.000 | 0.735 | 1.024 | 0.995 |
| **GLUTAMATE** | 1.000 | 0.735 | 1.024 | 0.995 |
| **MEVALONATE** | 1.000 | 1.130 | 1.017 | 1.477 |
| **SUCCINATE** | 1.000 | 2.157 | 1.016 | 0.868 |
| **2-DEOXY-D-GLUCOSE** | 1.000 | 0.947 | 1.009 | 0.227 |
| **GLUCURONATE** | 1.000 | 0.814 | 1.009 | 1.236 |
| **ITACONATE** | 1.000 | 1.129 | 1.007 | 1.047 |
| **GALACTARATE** | 1.000 | 1.157 | 1.003 | 1.067 |
| **GALACTURONATE** | 1.000 | 0.807 | 1.001 | 1.212 |
| **TYROSINE** | 1.000 | 1.548 | 0.996 | 0.842 |
| **HOMOSERINE** | 1.000 | 0.018 | 0.994 | 1.280 |
| **ALLOTHREONINE** | 1.000 | 0.018 | 0.994 | 1.281 |
| **N-ACETYLASPARAGINE** | 1.000 | 1.280 | 0.986 | 0.795 |
| **3-(3,4,5-Trimethoxyphenyl)propanoic acid** | 1.000 | 1.102 | 0.980 | 0.860 |
| **2-AMINOPHENOL-2** | 1.000 | 1.427 | 0.978 | 1.034 |
| **XANTHOSINE** | 1.000 | 1.247 | 0.977 | 0.902 |
| **PHENYLETHANOLAMINE** | 1.000 | 1.041 | 0.976 | 1.019 |
| **PYRIDOXINE-2** | 1.000 | 1.533 | 0.976 | 1.121 |
| **PYRIDOXINE-1** | 1.000 | 1.533 | 0.975 | 1.121 |
| **Dodecanoic acid** | 1.000 | 0.938 | 0.971 | 1.211 |
| **L-ORNITHINE** | 1.000 | 1.148 | 0.966 | 0.963 |
| **4-ACETAMIDOBUTANOATE** | 1.000 | 1.766 | 0.965 | 1.040 |
| **URACIL-1** | 1.000 | 1.194 | 0.964 | 0.922 |
| **4-PYRIDOXATE** | 1.000 | 2.121 | 0.961 | 1.134 |
| **2-METHYLMALEATE** | 1.000 | 0.978 | 0.949 | 0.897 |
| **TRYPTOPHAN** | 1.000 | 1.589 | 0.942 | 0.778 |
| **THREONINE** | 1.000 | 0.790 | 0.941 | 0.899 |
| **BETAINE** | 1.000 | 1.086 | 0.940 | 0.795 |
| **METHYLTHIOADENOSINE** | 1.000 | 1.120 | 0.936 | 0.889 |
| **GLYCOCHOLATE** | 1.000 | 1.915 | 0.932 | 0.935 |
| **N,N,N-TRIMETHYLLYSINE** | 1.000 | 0.985 | 0.927 | 1.009 |
| **D-ORNITHINE** | 1.000 | 1.230 | 0.924 | 0.946 |
| **UREA** | 1.000 | 0.994 | 0.922 | 1.288 |
| **METHIONINE-1** | 1.000 | 1.381 | 0.921 | 0.819 |
| **GLYCERATE** | 1.000 | 1.286 | 0.919 | 1.053 |
| **DIHYDROXYACETONEPHOSPHATE** | 1.000 | 0.493 | 0.913 | 0.629 |
| **Glyceraldehyde 3-phosphate** | 1.000 | 0.499 | 0.909 | 0.640 |
| **3-(2-HYDROXYPHENYL)PROPANOATE** | 1.000 | 0.978 | 0.908 | 1.544 |
| **ERYTHROSE-4-PHOSPHATE** | 1.000 | 0.589 | 0.907 | 1.292 |
| **TRIGONELLINE** | 1.000 | 0.943 | 0.906 | 0.945 |
| **Isovalerylcarnitine** | 1.000 | 1.468 | 0.901 | 1.140 |
| **PIPECOLATE** | 1.000 | 1.069 | 0.900 | 0.750 |
| **PHENYLALANINE** | 1.000 | 1.437 | 0.894 | 0.789 |
| **NICOTINATE-1** | 1.000 | 1.052 | 0.884 | 0.793 |
| **NICOTINAMIDE-2** | 1.000 | 1.403 | 0.884 | 0.907 |
| **ARGININE** | 1.000 | 1.136 | 0.882 | 0.918 |
| **HIPPURATE** | 1.000 | 1.941 | 0.880 | 0.895 |
| **METHIONINE SULFOXIDE** | 1.000 | 0.653 | 0.867 | 0.822 |
| **CREATININE** | 1.000 | 1.123 | 0.858 | 0.709 |
| **XANTHINE-2** | 1.000 | 1.683 | 0.856 | 0.814 |
| **O-ACETYLSERINE** | 1.000 | 0.761 | 0.849 | 0.789 |
| **PHENYLLACTATE** | 1.000 | 0.996 | 0.847 | 1.394 |
| **5-HYDROXYTRYPTOPHAN** | 1.000 | 1.707 | 0.845 | 0.695 |
| **PHOSPHOCREATINE** | 1.000 | 0.750 | 0.843 | 0.801 |
| **1-AMINOCYCLOPROPANECARBOXYLATE** | 1.000 | 0.880 | 0.838 | 0.883 |
| **RHAMNOSE** | 1.000 | 1.118 | 0.833 | 0.682 |
| **ISOLEUCINE** | 1.000 | 1.278 | 0.832 | 0.939 |
| **FOLATE** | 1.000 | 1.768 | 0.831 | 0.949 |
| **RAFFINOSE** | 1.000 | 1.188 | 0.829 | 0.923 |
| **DEOXYCARNITINE** | 1.000 | 0.739 | 0.814 | 1.076 |
| **HISTIDINE** | 1.000 | 0.931 | 0.812 | 0.836 |
| **METHIONINE-2** | 1.000 | 1.447 | 0.801 | 0.668 |
| **STACHYOSE** | 1.000 | 1.179 | 0.797 | 0.840 |
| **ASPARAGINE** | 1.000 | 0.456 | 0.796 | 0.677 |
| **ALLANTOIN** | 1.000 | 1.511 | 0.796 | 0.830 |
| **GLYCOCHENODEOXYCHOLATE** | 1.000 | 1.834 | 0.794 | 0.919 |
| **RIBOFLAVIN** | 1.000 | 1.917 | 0.792 | 0.893 |
| **BENZYLAMINE** | 1.000 | 1.147 | 0.791 | 0.814 |
| **URACIL-2** | 1.000 | 1.137 | 0.790 | 0.786 |
| **S-ADENOSYLMETHIONINE** | 1.000 | 1.087 | 0.786 | 0.450 |
| **2-HYDROXY-4-(METHYLTHIO)BUTANOATE** | 1.000 | 0.704 | 0.783 | 1.854 |
| **N-ALPHA-ACETYLLYSINE** | 1.000 | 1.166 | 0.782 | 0.627 |
| **N-ACETYLMETHIONINE** | 1.000 | 0.685 | 0.779 | 1.088 |
| **GLUCONATE** | 1.000 | 0.413 | 0.757 | 1.413 |
| **VALINE** | 1.000 | 1.152 | 0.741 | 0.787 |
| **4-GUANIDINOBUTANOATE** | 1.000 | 1.041 | 0.740 | 0.741 |
| **N-ACETYLASPARTATE** | 1.000 | 0.883 | 0.740 | 0.709 |
| **GLYCINE** | 1.000 | 0.806 | 0.739 | 0.839 |
| **GALACTITOL** | 1.000 | 0.882 | 0.730 | 1.035 |
| **MANNITOL** | 1.000 | 0.882 | 0.730 | 1.035 |
| **GLYOXYLATE** | 1.000 | 0.838 | 0.720 | 1.067 |
| **2-HYDROXYBUTYRATE** | 1.000 | 0.941 | 0.712 | 0.602 |
| **GLUTAMINE** | 1.000 | 0.859 | 0.697 | 0.584 |
| **HYDROXYPHENYLLACTATE** | 1.000 | 0.826 | 0.689 | 1.462 |
| **1-METHYL-L-HISTIDINE** | 1.000 | 1.061 | 0.688 | 0.376 |
| **3,4-Dihydroxyhydrocinnamic acid** | 1.000 | 0.824 | 0.686 | 1.451 |
| **Hydroxyphenyllactic acid** | 1.000 | 0.827 | 0.665 | 1.496 |
| **KYNURENATE** | 1.000 | 1.048 | 0.653 | 0.731 |
| **N-ACETYLALANINE** | 1.000 | 0.623 | 0.649 | 1.515 |
| **CARNOSINE** | 1.000 | 0.872 | 0.638 | 1.402 |
| **GLUTARYLCARNITINE** | 1.000 | 2.196 | 0.637 | 0.776 |
| **N-ACETYLGLYCINE** | 1.000 | 0.768 | 0.624 | 0.612 |
| **PROPIONYLCARNITINE** | 1.000 | 1.413 | 0.623 | 0.705 |
| **2-HYDROXYPYRIDINE** | 1.000 | 1.007 | 0.621 | 0.595 |
| **CITRULLINE** | 1.000 | 0.745 | 0.617 | 0.843 |
| **HEXOSE** | 1.000 | 0.815 | 0.617 | 1.475 |
| **GLUCOSAMINE 6-PHOSPHATE** | 1.000 | 0.325 | 0.614 | 1.020 |
| **ETHYLMALONATE** | 1.000 | 1.232 | 0.571 | 0.688 |
| **URIDINE DIPHOSPHATE GLUCOSE** | 1.000 | 1.098 | 0.562 | 0.749 |
| **CITRAMALATE** | 1.000 | 1.048 | 0.562 | 0.509 |
| **UDPgalactose** | 1.000 | 1.128 | 0.553 | 0.772 |
| **SALSOLINOL-2** | 1.000 | 1.541 | 0.547 | 0.747 |
| **N-ACETYLLEUCINE** | 1.000 | 1.059 | 0.536 | 0.625 |
| **O-PHOSPHOETHANOLAMINE** | 1.000 | 0.309 | 0.533 | 0.624 |
| **ETHANOLAMINE PHOSPHATE** | 1.000 | 0.310 | 0.532 | 0.627 |
| **BETA-ALANINE** | 1.000 | 0.399 | 0.519 | 0.794 |
| **SERINE** | 1.000 | 0.516 | 0.497 | 0.600 |
| **SARCOSINE** | 1.000 | 0.402 | 0.492 | 0.771 |
| **URIDINE DIPHOSPHATE-N-ACETYLGLUCOSAMINE** | 1.000 | 1.069 | 0.484 | 0.836 |
| **URIDINE DIPHOSPHATE-N-ACETYLGALACTOSAMINE** | 1.000 | 1.078 | 0.474 | 0.844 |
| **ANSERINE** | 1.000 | 0.950 | 0.459 | 0.873 |
| **N-FORMYL-L-METHIONINE** | 1.000 | 0.327 | 0.448 | 1.210 |
| **SALSOLINOL-1** | 1.000 | 1.082 | 0.420 | 0.762 |
| **cis-ACONITATE-1** | 1.000 | 0.727 | 0.399 | 0.498 |
| **PHOSPHOSERINE** | 1.000 | 0.473 | 0.396 | 0.670 |
| **N-ACETYLPUTRESCINE** | 1.000 | 0.360 | 0.384 | 0.342 |
| **PHENYLACETATE** | 1.000 | 0.883 | 0.383 | 1.421 |
| **N-ACETYLPHENYLALANINE** | 1.000 | 1.005 | 0.382 | 0.487 |
| **FUMARATE** | 1.000 | 0.490 | 0.379 | 0.935 |
| **MALEATE** | 1.000 | 0.489 | 0.369 | 0.905 |
| **SORBITOL** | 1.000 | 0.540 | 0.366 | 1.144 |
| **MALATE** | 1.000 | 0.412 | 0.336 | 0.898 |
| **O-ACETYLCARNITINE-2** | 1.000 | 0.685 | 0.315 | 0.275 |
| **L-ALANINE** | 1.000 | 0.299 | 0.311 | 0.443 |
| **D-ALANINE** | 1.000 | 0.294 | 0.309 | 0.438 |
| **OPHTHALMATE-2** | 1.000 | 0.670 | 0.301 | 0.448 |
| **DIMETHYLGLYCINE** | 1.000 | 0.730 | 0.300 | 1.356 |
| **CITRATE** | 1.000 | 0.712 | 0.300 | 0.449 |
| **PROLINE** | 1.000 | 0.802 | 0.281 | 0.588 |
| **GAMMA-AMINOBUTYRATE** | 1.000 | 0.185 | 0.241 | 1.302 |
| **PORPHOBILINOGEN-1** | 1.000 | 1.162 | 0.214 | 0.949 |
| **O-ACETYLCARNITINE** | 1.000 | 0.388 | 0.213 | 0.266 |
| **PHOSPHORYLCHOLINE** | 1.000 | 0.093 | 0.196 | 0.338 |
| **GUANIDINOACETATE** | 1.000 | 0.165 | 0.176 | 0.070 |
| **ASPARTATE** | 1.000 | 0.128 | 0.139 | 0.235 |
| **CYSTINE** | 1.000 | 0.663 | 0.005 | 0.005 |
