# Supplementary material for: Mitochondrial fatty acid synthesis coordinates oxidative metabolism in mammalian mitochondria
Source: eLife. 2020 Aug 17;9:e58041. doi: 10.7554/eLife.58041 (PMC7470841; doi:10.7554/eLife.58041)
Supplement: Supplementary file 2. — Relative expression of proteins associated with skeletal muscle differentiation from SL-TMT experiment. Values are arbitrary units. [file elife-58041-supp2.docx]

**Supplementary File 2:**

| **Gene Symbol** | **Description** | **GFP-1** | **GFP-2** | **Oxsm-1** | **Mecr-1** | **Mecr-2** |
| --- | --- | --- | --- | --- | --- | --- |
| Cdh15 | CAD15_MOUSE Cadherin-15 | 14.80 | 12.63 | 5.22 | 8.53 | 5.35 |
| Myod1 | MYOD1_MOUSE Myoblast determination protein 1 | 12.88 | 10.20 | 5.15 | 9.85 | 7.56 |
| Tnnt3 | TNNT3_MOUSE Troponin T, fast skeletal muscle | 23.46 | 11.06 | 3.71 | 6.21 | 3.24 |
| Tnni2 | TNNI2_MOUSE Troponin I, fast skeletal muscle | 26.59 | 9.76 | 2.73 | 6.25 | 2.42 |
| Tnnt2 | TNNT2_MOUSE Troponin T, cardiac muscle | 29.13 | 8.47 | 2.11 | 6.04 | 2.08 |
| Tnni1 | TNNI1_MOUSE Troponin I, slow skeletal muscle | 29.93 | 10.61 | 1.46 | 4.71 | 1.33 |
| Tnnc1 | TNNC1_MOUSE Troponin C, slow skeletal and cardiac muscles | 32.32 | 11.36 | 0.78 | 3.76 | 0.77 |
| Myh1 | MYH1_MOUSE Myosin-1 | 29.34 | 13.57 | 2.80 | 2.02 | 1.56 |
| Mb | MYG_MOUSE Myoglobin | 32.32 | 11.36 | 1.44 | 2.31 | 1.41 |
| Chdh | CHDH_MOUSE Choline dehydrogenase, mitochondrial | 19.97 | 25.70 | 1.09 | 1.60 | 1.28 |
| Cd44 | CD44_MOUSE CD44 antigen | 17.76 | 13.15 | 4.69 | 5.60 | 6.53 |
| Mylpf | MLRS_MOUSE Myosin regulatory light chain 2, skeletal muscle isoform | 33.08 | 9.48 | 2.23 | 2.60 | 1.62 |
| Myh3 | MYH3_MOUSE Myosin-3 | 33.53 | 9.34 | 2.91 | 2.11 | 1.30 |
| Acta1 | ACTS_MOUSE Actin, alpha skeletal muscle | 29.41 | 11.38 | 1.84 | 3.74 | 2.61 |
| Myl1 | MYL1_MOUSE Myosin light chain 1/3, skeletal muscle isoform | 29.19 | 12.41 | 2.05 | 3.13 | 1.92 |
| Myl4 | MYL4_MOUSE Myosin light chain 4 | 21.07 | 8.25 | 8.05 | 4.75 | 5.17 |
| Myl6b | MYL6B_MOUSE Myosin light chain 6B | 28.33 | 7.25 | 2.69 | 5.25 | 4.18 |
| Sorbs2 | SRBS2_MOUSE Isoform 2 of Sorbin and SH3 domain-containing protein 2 | 30.69 | 13.23 | 2.43 | 1.57 | 1.34 |
| Csrp3 | CSRP3_MOUSE Cysteine and glycine-rich protein 3 | 32.20 | 11.30 | 1.17 | 3.02 | 1.01 |
| Nrap | NRAP_MOUSE Nebulin-related-anchoring protein | 25.07 | 15.64 | 2.70 | 3.72 | 0.45 |
| Klhl41 | KLH41_MOUSE Kelch-like protein 41 | 19.74 | 12.08 | 5.16 | 5.67 | 4.70 |
| Ccdc141 | A2AST1_MOUSE Coiled-coil protein associated with myosin II and DISC1 | 27.08 | 13.57 | 2.55 | 3.18 | 1.65 |
| Neb | A2AQA9_MOUSE Protein Neb | 25.63 | 10.39 | 3.78 | 4.87 | 3.42 |
| Bin1 | BIN1_MOUSE Myc box-dependent-interacting protein 1 | 22.25 | 11.53 | 5.00 | 5.80 | 4.14 |
| Ckm | KCRM_MOUSE Creatine kinase M-type | 20.99 | 13.62 | 3.52 | 6.25 | 3.63 |
| Ckb | KCRB_MOUSE Creatine kinase B-type | 23.97 | 13.29 | 2.69 | 3.22 | 4.93 |
| Ank1 | ANK1_MOUSE Isoform Mu7 of Ankyrin-1 | 19.68 | 22.43 | 0.98 | 4.80 | 0.80 |
| Trim72 | TRI72_MOUSE Tripartite motif-containing protein 72 | 27.22 | 10.64 | 3.59 | 4.78 | 2.27 |
| Itga7 | ITA7_MOUSE Integrin alpha-7 | 17.66 | 13.34 | 5.37 | 5.96 | 4.66 |
| Akap6 | E9Q9K8_MOUSE Protein Akap6 | 19.39 | 14.68 | 4.31 | 5.04 | 3.17 |
| Dusp27 | DUS27_MOUSE Inactive dual specificity phosphatase 27 | 15.61 | 14.64 | 5.26 | 6.56 | 3.95 |
